# Factors impacting the access and use of formal health and social services by caregivers of stroke survivors: an interpretive description study

**DOI:** 10.1186/s12913-022-07804-x

**Published:** 2022-04-01

**Authors:** Anna Garnett, Jenny Ploeg, Maureen Markle-Reid, Patricia H. Strachan

**Affiliations:** 1 Arthur Labatt Family School of Nursing, Western University, London, ON Canada; 2 School of Nursing, McMaster University, Hamilton, ON Canada; 3 Aging, Community and Health Research Unit, McMaster University, Hamilton, ON Canada; 4 McMaster University, 1280 Main St. W, Hamilton, ON Canada

**Keywords:** Stroke, Caregivers, Health services, Social services, Access, Use

## Abstract

**Background:**

Evidence has shown that family and friend caregivers of stroke survivors are significantly and negatively impacted by caregiving. The negative effects of caregiving may persist over time suggesting that caregivers might benefit from ongoing engagement with supportive services. However, little is known about caregivers’ use of formally funded health and social services, or the factors influencing their access to and use of these services. The aim of this study is to increase understanding of the factors that influence stroke caregivers’ access and use of formal health and social services, from the perspective of stroke caregivers and healthcare providers.

**Methods:**

A qualitative study was conducted with stroke caregivers and health providers in Ontario, Canada using interpretive description. In-depth interviews were conducted with caregivers of survivors who experienced a stroke between six months to five years previous and healthcare providers who support caregivers and stroke survivors. All participants provided written informed consent. Interview data were analyzed using constant comparison to identify codes and develop key thematic constructs.

**Results:**

A total of 40 interviews were conducted with 22 stroke caregivers at an average 30-months post-stroke and 18 health providers. Factors that influenced stroke caregivers’ access and use of services included: finances and transportation; challenges caregivers faced in caring for their health; trust that they could leave their family member and trust in health providers; limited information pertaining to services and a lack of suitable services; and the response of their social networks to their caregiving situation.

**Conclusion:**

Stroke caregivers experience significant challenges in accessing and using formal health and social services. These challenges could be addressed by increasing availability of subsidized community-based supports such as respite and counselling tailored to meet the ongoing needs of caregivers. Systemic change is needed by the health system that readily includes and supports caregivers throughout the stroke recovery continuum, particularly in the community setting.

**Supplementary Information:**

The online version contains supplementary material available at 10.1186/s12913-022-07804-x.

## Background

Experiencing a stroke can be a profound and life altering experience, not only for the stroke survivor but also for their family and friend caregivers (hereafter referred to as caregivers). It is estimated that each year approximately 62,000 Canadians will experience a stroke [1]. Of these strokes, 5.1% (3,162) will lead to death, but 58,838 Canadians are expected to survive [2]. In 2018, approximately 405,000 Canadians were living with the effects of stroke, including various disabilities related to mobility, physical independence, and social functioning [1]. Stroke recovery may also be complicated by the presence of other chronic conditions [3]. Up to 75% of stroke survivors have three or more comorbidities complicating their recovery and resulting in the need for ongoing care [4]. Hypertension is both a risk factor for stroke, and the most commonly occurring comorbidity in people who have survived a stroke [5, 6]. Other multiple chronic conditions (MCC) commonly reported in conjunction with stroke include arthritis, asthma, mood disorders and/or anxiety, hyperlipidemia, and diabetes [7, 8]. The co-occurrence of these conditions with stroke can result in the need for: complex medication regimes, multiple specialist consults and increased challenges with self-care [9]. After receiving treatment in acute and rehabilitation settings, approximately 80% of stroke survivors will return home after receiving treatment in acute and rehabilitation settings where caregivers will provide the majority of required support [10, 11].

Stroke survivors frequently require longer-term support from caregivers in a community setting due to ongoing impairment [12–16]. Although these stroke survivors may receive services such as outpatient rehabilitation and community-based programs, caregivers continue to provide most of the daily care with 61–91% of caregivers providing support with basic and instrumental activities of daily living at 12 months post-stroke [12, 17]. Furthermore, stroke caregivers provide a median of 35 h of care per week to stroke survivors in the first year following stroke [17].

While providing ongoing assistance to a stroke survivor can be rewarding, it can have negative impacts on the health and well-being of the caregiver [18]. Extensive literature outlines these negative impacts on outcomes such as psychological health (e.g., burden, stress and anxiety), social impacts (e.g., relationships with friends, and family members), and finances and employment [18–24]. For example, prolonged (> 6 months) caregiving has an association with increased depression, anxiety, decreased cognitive function, decreased quality of life, physical strain, exhaustion, and even mortality in caregivers [18].

Acknowledgement of the importance of caregiver roles in health provision is increasing and with this has come the recognition that they must be included in the circle of care for stroke recovery [25]. Throughout the caregiving journey, caregivers’ use of services such as counselling and respite may support them to care for their own health. Formal health and social services, defined as those services that directly support caregivers or indirectly affect the caregiver by providing services to the stroke survivor, have the potential to support stroke caregivers resulting in improved health and quality of life. Research demonstrates that caregivers use formal health and social services (e.g., general practitioners, community nurses, counselling, and day centres) even after a considerable time has passed since the stroke [26, 27]. Furthermore, research also indicates that caregivers who engaged in service use, were generally satisfied with the services they utilized [27]. For example, when caregivers used a formal family support program (starting within six weeks post-stroke) researchers found that caregivers receiving the program had greater improvements in physical health, mental health and quality of life at six months post-stroke compared with caregivers receiving usual care [26, 28].

However, not all stroke caregivers have access to or make use of these community services [22, 27, 29]. The literature suggests that services are used infrequently despite being rated as important are respite, stress management, and conversing with peers [26, 27]. Furthermore, despite stroke caregivers’ need for ongoing support, only 5% to 19% of caregivers use respite care [26, 27]. Research suggests that caregivers and stroke survivors’ service access and use is influenced by a variety of circumstances including demographic factors such as age and gender [26, 27, 30–32], caregivers’ knowledge of services [26, 27, 29, 33, 34], social-relational factors such as marginalisation of caregivers [33, 34], service provision and accessibility factors such as type of available services [29, 34, 35] and stroke survivor-related factors [26, 30, 32, 36].

While there is some available knowledge of factors that impact stroke caregiver use of supportive services across three continents, little is known about the Canadian context [37]. Moreover, gaps exist in our understanding why caregivers make limited use of services like respite or counselling, despite finding them important for their health and well-being. Furthermore, there is a need to explore and understand the factors impacting caregivers’ service access and use over a long-term perspective, something this is limited in the current literature.

A qualitative study using interpretive description (ID) is ideally positioned to inform a deeper understanding of service use behaviour by stroke caregivers through its emphasis on exploring individual experiences, grounding in health disciplines and use of multiple sources of data to provide triangulation of studied phenomena [38, 39]. This information can be used to inform stroke-related policy that recognizes the caregiver role and results in the delivery of services and programs that better support stroke caregivers in their caregiving role. Given the increasing prevalence of stroke, the increases in caregiver burden and the desire to support stroke survivors at home, the importance of effectively supporting caregivers will only increase with time [1, 40–42]. Therefore, the purpose of this study was to explore the factors that impact caregivers’ (of community dwelling stroke survivors) access and use of formal health and social services within a Canadian context.

## Methods

### Study design and setting

This study was conducted in various southern Ontario communities using Thorne’s qualitative interpretive description (ID) approach [38]. Studies conducted using ID embrace the contextual nature of human behaviour and with its interpretive elements make it well-placed to explore multi-faceted clinical issues with a rigorous, well-established methodological approach that is both informed by and can inform clinical practice [43]. The inclusion of health providers was consistent with ID’s emphasis on the value of the thoughtful clinician perspective [38]. Our approach includes patients, caregivers, and healthcare providers as study participants which enabled triangulation of the data and provided alternative perspectives grounded across time and context, thus helping to ensure the analytic rigor of the study findings [44].

### Theoretical framework

An adaptation of Grembowski et al.’s (2014) conceptual model of the role of complexity in the care of patients with MCC guided interview questions and analysis [45]. This framework was chosen because of its emphasis on a broad range of health, social and contextual factors that influence the person and their caregiver and their interactions with the health system.

The Role of Complexity in the Care of Patients with Multiple Chronic Conditions (RC – MCC) framework provides a relational perspective on the patient or caregiver, the health system and the socio-economic factors, which exert influence on the interactions between caregivers and the healthcare system [45]. The RC – MCC framework was used to inform the questions that guided the in-depth interviews with stroke caregivers and health providers and for the interpretation of study findings.

### Eligibility

Eligible caregivers who met the following inclusion criteria were enrolled in the study: (a) family or friend caregivers over the age of 18 years who had experience providing support to a stroke survivor in the community setting, and (b) English-speaking. The caregiver was defined as a family member (e.g. spouse, child, parent) or friend who had experience providing physical or emotional support to a stroke survivor. Those caregivers who only had experience with providing support to a stroke survivor who was residing in alternative level of care such as inpatient rehabilitation, acute care hospital or long-term care were ineligible to participate in the study. Stroke survivors were required to be over the age of 18 and to have experienced their stroke at least six months and no more than five years ago.

Eligible health providers employed in hospital and community settings and who met the following inclusion criteria were enrolled in the study: (a) provided direct or indirect care to the stroke survivor and/or stroke caregiver and (b) provided or were able to provide information and /or education regarding community-based services for a stroke caregiver.

### Sample

Stroke caregivers and health providers from various communities in Southern Ontario, Canada were sampled between April and September 2017. A combination of four purposive sampling strategies were used: criterion sampling, maximum variation sampling, theoretical sampling and snowball sampling [38]. In the current study, reference to other studies’ sample size served as a starting point for determination of our sample size. A search of the literature showed a range between eight to 18 participants with an average sample size of 12 was acceptable for interpretive description studies [46–51]. Additionally, data collection and analysis were iterative enabling us to begin identifying codes and broad themes as data collection proceeded. This helped us to identify and address remaining data needs to enrich aspects of analysis that had remained underdeveloped. Ultimately data collection concluded when the researcher judged that further data collection would not significantly deepen understanding of the phenomenon [38, 39, 52].

### Recruitment & data collection

**Fig. 1:**
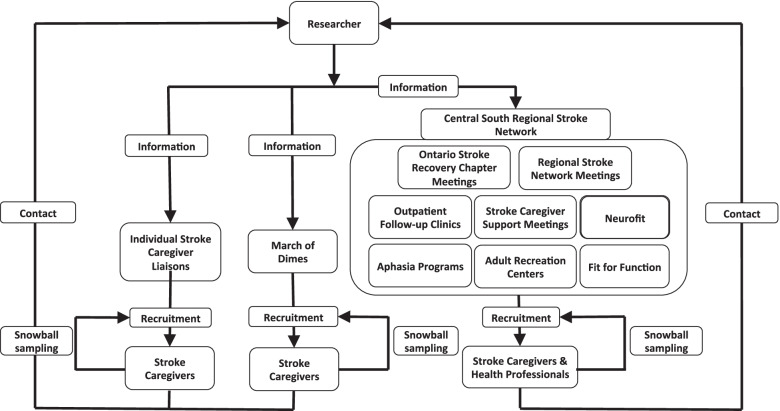
Recruitment strategy. This figure illustrates how the recruitment of stroke caregivers and health providers proceeded

The initial stroke caregiver recruitment strategy involved distributing study information cards, posters, and introducing the study to stroke caregivers to the partner organizations of the Central South Regional Stroke Network such as Ontario Stroke Recovery Chapters, follow-up clinics for stroke survivors, Regional Stroke Network meetings and March of Dimes meetings. A research coordinator at the Central South Regional Stroke Network also distributed study information cards and posters, which facilitated recruitment of health providers. The primary investigator (PI) distributed study information at meetings associated with a variety of partner organizations (day programs, aphasias programs, YMCA Fit for Function programming) which helped to recruit both caregivers and healthcare providers (Fig. 1). Additionally, the PI worked to develop relationships with a broad group of health providers who worked with stroke survivors and their caregivers which made it possible to distribute study information cards to other potential stroke caregiver participants. Prior to recruitment into the study, the PI explained the purpose of the study, her background and her role in the research to any potential study participants via an in-person meeting or by telephone. There were no prior relationships with any study participants. The PI’s interest in the topic including potential biases (e.g. perspective as health provider) were also disclosed to participants.

In-depth interviews were conducted in-person or by phone with all study participants by a female doctoral student who was also a RN, MSc (AG) with extensive experience working in a variety of community and research settings and who had advanced training in qualitative and quantitative research methods. Interview questions were developed using the findings of a literature review as well as the RC-MCC framework and piloted with one stroke caregiver and one stroke survivor [45, 53]. After piloting the questions, the findings were discussed with members of the research team (AG and JP) and revised to ensure adequate richness of information attainment. Repeat interviews were not conducted. Interviews took on average 60 min, were audio-recorded, de-identified and transcribed prior to data analysis. The interview process entailed collecting basic demographic information, followed by open-ended questions specific to the participant being interviewed (stroke caregivers or health providers). Questions were related to: (a) the caregiving role, (b) social support, (c) the stroke survivor, (d) health system and community resource access, use and provision, (e) the need for service, and (f) the caregiver’s health (Appendix A). Additional clinical data were collected about the stroke survivor, including the date of the patient’s stroke, the type of stroke, including health challenges and the number of chronic conditions to further contextualize the caregivers’ role.

Data collection took place in participants’ homes, in community settings (e.g., day program) and via telephone with either a family member present or with just the researcher. The process of data collection was enhanced through the use of ecomaps which involved the development of a pictorial representation of the inter-relationships between caregivers, stroke survivors and health providers as well as community-based services [54] (Appendix B). The visual representation of the data was then corroborated with interview data to provide a more in-depth representation of the study findings [55, 56]. Additionally, field notes were kept throughout the study and served as an audit trail. Study quality and rigor was ensured through the use of techniques such as: (a) representative credibility, (b) contextual awareness, and (c) analytic logic, an expectation that there is a clear logical flow of reasoning and decision-making [44].

### Data analysis

Data collection and analysis occurred concurrently, with broad thematic patterns identified using ecomaps and the interviewer reflections on the transcripts [57]. For interpretive description analysis, guidance from Thorne, Saldana, and Morse provided direction for use of codes, coding techniques, and cognitive processing [38, 58, 59].

Transcripts were re-read several times by the PI and initial impressions were added to the data sheets in accordance with Morse [59]. The visual representation of the data collected using ecomaps was then corroborated with the interview data to provide a more in-depth representation of the study findings [55, 56]. The PI then provided a subset of transcripts to each member of the research team (PG, MM and PS). PG, MM and PS independently reviewed the same subset of the transcripts. The PI also reviewed that subset of transcripts and then all members of the research team came together to discuss first impressions, key findings and to begin to identify codes. This collaborative process continued throughout the coding and thematic development stages of the analysis. The preliminary coding list and any areas of broad discrepancy in coding were discussed with the research team and resolved before progressing to detailed coding using computer software. Next, coding was conducted using NVivo 12.0.0, [60] and guidance from Saldana [58] with a step-wise process known as first and second cycle coding. First cycle coding entailed using simple, explicit codes to categorize the data (e.g., transportation).

After initial coding, second cycle coding took place where codes identified in the first cycle of coding were grouped according to a concise list of themes [58]. Constant comparative analysis was used to compare coded segments of one transcript with other caregiver and health provider transcripts. Consistent with the thoughtful clinician perspective [38], data from health providers was used to corroborate or contrast the experiences of stroke caregivers.

Ethics approval for this research was obtained from the Hamilton Integrated Research Ethics Board and written informed consent was obtained from all participants (REB 2770).

## Results

A total of 40 individuals participated in the study (22 caregivers and 18 health providers) (Table 1 and 2). No participants approached during recruitment declined to participate and none dropped out at any stage of the study. Participating caregivers were an average of 62.9 years (*SD* = 9.7) and about two thirds (63.6%) were female. The mean length of time spent in a caregiving role was 30 months (*SD* = 14.0 months). The caregiver participants cared for a total of 21 stroke survivors (Table 3). The average age of the stroke survivors was 63.4 years (*SD* = 12.1), and 61.9% were male. Participating health providers practiced across a range of clinical settings (Table 4). Just over one half (55.5%) of the providers had been practicing in their respective professions for 16 or more years, and one-half had practiced in their current position for three years or less.

Through analysis we identified five major themes pertaining to factors that impacted caregivers’ access and use of formal health and social services: (a) finances and transportation, (b) challenged to take care of my own health, (c) trust, (d) limited information and lack of suitable services, and (e) social support networks (Table 5).

### Finances and Transportation – “You have to pay, well we can’t afford $50 or $75 twice a week”

Finances were an important factor that had notable bearing on caregivers’ ability to access and use services. One caregiver who was asked if he paid out-of-pocket for any services for either himself or the stroke survivor, responded with:

Okay I take her to [a day program]. So, we pay for that. I take her to a group physiotherapy exercise program once a week, so we pay for that. I take her to a foot care specialist chiropodist, so I pay for that. (SCG 20)

The caregiver in this example had taken early retirement to care for his wife and was managing her care on a fixed income. Some services and programs were subsidized, but the cumulative nature of the costs quickly depleted their monthly budget. Other caregivers echoed these financial worries and constraints on their ability to use programs and services. One caregiver said, “you’re looking at $25 a day [to attend a day program], you know. I think how you are going to support it, wow.” (SCG 08) This caregiver was referring to the cumulative costs of attending day programs, which included transportation, program costs and lunch. In that situation, the stroke survivor, who was in her early fifties, was unable to work and her spouse, her primary caregiver, was also unable to work because of cancer and its associated treatment.

Reductions in income and employment were commonly discussed by many participants. One caregiver commented, “yeah and he made good money cause he's a truck driver, so I didn't have to work. Yeah [he had to leave his job].” (SCG 09) Providers also identified that out-of-pocket expenditures influenced service use. They noted that the costs associated with attending programs constrained service use and they commented on the paucity of available funding streams to support caregivers and stroke survivors. For example, one provider commented:

Well, so, those ones [caregivers], must be very careful about every penny they spend. Coming to our day program or aphasia program there is a cost. And some of them do limit it; limit their attendance because of the affordability. And we’re pretty cheap. We’re six bucks for a half day. But if you’re on Ontario Disability Support Program…It’s gone pretty fast. Six bucks a week, yeah and then on top of that it’s the transportation, is another six bucks each way. So that’s an $18 day for half a day. Half a day. So that’s a big deal. It’s a real big deal. (HP 04)

Health providers and caregivers alike identified significant challenges in navigating supportive financial assistance which included having to complete large amounts of paperwork to access funding for equipment or to be reimbursed by insurance companies for costs they had already incurred. One caregiver indicated that:

My finances, trying to negotiate the myriad of government agencies has been a challenge and I've learned who to contact about things like disability tax credit and stuff like that. (SCG 20)

A health provider’s summation of caregivers ability to manage over time was explained in this impactful quote: “You know I think there are two major determinants. There’s the health of the caregiver and how much money they have. I don’t think there’s ever enough money to provide all the support.” (HP 08) The health provider’s comments capture the importance of finances in enabling caregivers and their care recipients to assess health and social service that will support their caregiving role.

While subsidized transportation can help facilitate access to formal services, not all people have access to this benefit. For example, when talking about potential services to transport her husband, one caregiver responded, “ah, distance yes. I wish I could get a service, a drive to [husband]’s day program, but apparently we’re in a dead zone or something.” (SCG 06) She was referring to the fact that she lived in a rural area that fell on the border between two jurisdictions, which complicated gaining access to services such as DARTS (Accessible Transportation Service) or Wheel Trans to assist with transportation. Furthermore, using public transport or even subsidized transport was not an option for all stroke survivors. For example, another caregiver spoke about the challenges she faced in getting transportation services for her husband:

We got him into [program], and then [name] is going to help drive him, and stuff like that because he didn’t have transportation and I also had to be at work, here in [location], and he didn’t qualify for, what is it? Wheel Trans, or whatever….

Because he was ambulatory, and he could walk the 75 m or whatever the assessment is but cognitively, he’d walk out in front of a car. (SCG 10).

In that situation there were services available, but the stroke survivor’s circumstances of being physically able though having a cognitive handicap meant that they could not take advantage of them. In this instance, the caregiver was left struggling to find an alternative form of transportation, which often did not exist, or meeting the transportation requirements themselves. This caregiver’s experience was echoed by a provider:

So, I mean in terms of efficiency for the patient and family, not always ideal. And also, a patient has to be cognitively able to ride alone. Right. It’s very rare, there are I think exceptions where I think, like if a spouse or family can go with the patient, but there’s always a big question around can the patient ride alone. And again, a huge part of our population is not able to ride alone. (HP 03).

This provider had extensive experience with stroke survivors and those who had experienced traumatic brain injuries. She pointed out how people with cognitive limitations can end up being marginalized by the constraints of the services available.

### Challenged to take care of my own health – “How can I find the time?”

Caregivers and providers noted that limited time and coping ability hindered caregivers’ ability to use services to support their health. One health provider recounted a tragic situation where the caregiver was neglecting her health:

I was doing home visits with them because her health was really going downhill. She had terrible pain…Like she wasn’t moving off the sofa, and the guy was in a wheelchair and... I said, “Look you have got to go see the doctor and have them investigate this back pain” … She neglected herself. So, it ended up that she was full of cancer, and she died… then he went into a retirement home then he died. (HP 04)

Another contributing factor to the difficulty caregivers experienced in taking care of their own health was that there were few services available such as respite that would give caregivers the valuable time and space to engage in health supporting behaviours. In many instances respite was available once per week and that time was allocated to attend to tasks of daily living such as banking, groceries, and other household chores. One caregiver noted, “you know my three hours is spent going multiple places and shopping and doing other things like car appointments.” (SCG 20).

Constraints in the health system and limited system wide attention on the importance of caregiver health were integral in influencing caregivers’ ability to take care of their health and use supportive services. Health providers talked about the gradual decline in health that can affect caregivers over the long-term, “they get depressed. They get anxious. They’re worried all the time, about their loved one and their health suffers.” (HP 05) Providers clearly identified and understood the difficulties caregivers were facing but they acknowledged that the solutions such as increased respite, improved advocacy for caregivers and inclusion of the caregiver in the circle of care were difficult to achieve within the current health and social context. One provider remarked:

The take care of yourself is a, it's a superficial comment. On the other hand, it's difficult to learn enough about the caregiver sort of medications and whatnot to ...We can't be the caregiver's doctor. (HP 14)

These health providers could not supply the services that caregivers needed. But the caregivers also didn’t have the time or resources to access the services so often they went without.

### Trust—“He’s breathing, but is that good enough?”

A common experience among caregivers was their difficulty trusting that if they left their loved one alone, they would still be all right when they returned home. In some instances, the caregiver had a negative experience that eroded their ability to trust, for example one caregiver recounted:

When he had his first stroke… I said to him “(husband) I’m just going to run to the store. I’ll be gone for just a few minutes.” I came home, like honest to God, fifteen minutes later, he’s in the kitchen, he’s got a tie kind of half on over his pyjamas… He thinks he’s getting ready to go to work, and he’s making breakfast… I said, “what are you doing?” And he said, “I’m making breakfast for us.” I said (Husband), “it’s 8 o’clock at night.” … and he said, “but don’t worry I’ve already taken my morning meds.” (SCG 04)

The caregiver’s ability to trust in safely leaving her loved one was challenged on many levels. Not only had the stroke survivor confused the time of day but he had also engaged in two potentially life-threatening activities: (a) taking his morning medications at night, and (b) his struggles in using an appliance that could potentially be a fire hazard.

Another caregiver described her concerns about trusting that she could safely leave her loved one alone, particularly at night:

But my fear as far as sleeping at night, it's a little bit better now. It's not as bad, but every once in a while you'll get this well, I wonder if he's okay. But you know you're watching and yeah, he's breathing but is that good enough? Because he isn't going by himself. Because that's just not the way it's going to be right? If he's going to die, I want to be there when he does. (SCG 14).

The caregiver in this instance had an extremely unsettling experience when her spouse first had his stroke. He had the stroke while sleeping such that the caregiver did not recognize the signs of stroke and her spouse went untreated for a long period of time. This experience was extremely traumatic for the caregiver and although her husband had recovered to a great extent, she still could not trust that he would be all right in her absence. Although the caregiver was determined that her life would return to normal, she was left with lingering trust issues about her husband’s safety. She did not confide this fear to a health provider and essentially suffered in silence.

Caregivers also struggled to trust in the providers who were caring for the stroke survivor. If the caregiver did not trust the service provider, they were less likely to use the services and were, therefore unable to benefit from the respite that these services could provide. Through one example it became clear that support groups designed to help caregivers, were more akin to a luxury not a priority, and frequently underutilized as one caregiver said, “[I’m] not able to go to the support group…Because of the fact I can't get support, trusted support for [Name] at the same time to do that.” (SCG 20).

One health provider indicated that respite is only effective if the caregiver trusts the quality of the service. For example, one provider described:

If you’ve got that personal support worker or whoever coming into their home and they’re still not 100% comfortable with that person, and they don’t take that time to build that rapport, that respite time isn’t… they’re going to spend it worrying. They [caregivers] are going to spend the whole time at the grocery store worrying, or they’re not going to be able to actually get something out of that. (HP 06)

In interpreting the above quote, it is possible that a caregiver’s lack of trust or comfort in using respite services could lead the caregiver to worry more rather than having the planned break that the service was intended to provide.

### Limited information about available services and lack of suitable services—“There’s a year-long wait list”

Most caregiver participants indicated that they had insufficient information about available services and how best to access them during their caregiving journey. One caregiver explained:

You know and these people are professionals it's like you know when you start a job at a factory nobody really wants to tell you [that you] have to learn that and that's what I found, I had to learn everything. And you know people oh my God they just tell you “oh well you can get disability.” No, you can’t. (SCG 09).

Not only did the caregivers experience challenges in accessing services that they knew about such as disability, they also struggled to find out what other services were available to support them. For example, “we didn’t know, we didn’t know if there was any help out there because nobody said there was any help out there.” (SCG 15) And, “I just want to back up and say that when I was at home looking for help, I was able to sit on the computer and try and find things. And that was the whole issue, trying to find things was very, very difficult.” (SCG 02) Providers interviewed were only familiar with stroke navigators in an inpatient setting, “the stroke navigators that I’ve known about they’re usually just for hospital [not community].” (HP 04) Another provider spoke about the value of having a resource person for caregivers to contact for information on availability and accessing services, “Some of it is also people don’t know what’s out there, and they don’t know what questions to ask. I think it would be really helpful if people had someone to call.” (HP 02).

Stroke caregivers expressed frustration about the lack of services available to meet their needs and the needs of the stroke survivor. Caregivers early in their caregiving journey were often looking for guidance on how to manage specific situations with the stroke survivor. As one caregiver said:

And I just kind of sat there and blubbered away and cried. “What do you guys do?” “Oh, well, I just you know…” No specific guidelines. I needed more yeah. And I don’t know whether I would have got it from a professional person. But if it was a professional person, if I ever had to go to a professional person again, I would love to speak to somebody who is familiar with the stroke situation. (SCG 04).

The caregiver was referring to her experience in consulting with a caregiver support group and a counsellor. In the case of the caregiver support group, she felt the group was more geared to socializing and less towards providing direct caregiver support, something she needed at that point in time. In the case of the counselling, she wanted concise, focused support to cope with the acute stress brought on by caregiving for a spouse who had significant cognitive deficits. The caregiver felt that her time and energy were wasted trying to explain her situation to the counsellor. She believed that someone more familiar with stroke would have understood her position at the outset thus enabling her to focus on her needs rather than requiring lengthy pre-emptive explanations.

Health providers discussed issues with the lack of follow-up with the caregiver in the community setting. For example, one health provider discussed a specific situation as follows:

So, the couple that I told you about that had the dynamic pre-existing to the stroke and that, were probably financially challenged and challenged in other ways, that particular caregiver really could have used some ongoing support…And that wasn’t there for her. (HP 02).

This health provider suggested that the caregiver would likely be struggling when the stroke survivor returned to the community but acknowledged that the system was unable to reach out to the caregiver.

### Social support networks – “They deserted us; they just don’t come around”

The availability of informal support influenced caregivers’ access and use of formal health and social services. Caregivers described that their social networks were either avoidant or that their social networks rallied to support them. Several caregivers exhibited sadness and dismay as they explained how they felt they were abandoned or avoided by friends who had previously been central pillars in their lives after their loved one’s stroke. For example, one caregiver said, “they deserted us. They just don’t come and visit. And I just don’t know if they know how to deal with this or not, I don’t know. No, a lot of people they just don’t come around.” (SCG 08) Another caregiver said, “Friends were scared. They didn’t want to deal with it.” (SCG 04) The caregivers alluded to the difficulties that some people may have had in relating to the stroke survivor especially if they had aphasia or pronounced physical or cognitive deficits. This perspective was echoed by another caregiver who said, “it seems that most friends are fair-weather friends if you want to call it that. A lot of friends don't know what to do, don't know what to say, so they just don't.” (SCG 20).

A feeling of social isolation although common was not ubiquitous among all caregivers who participated in the study. Some caregivers described how their social networks rallied to support them in their caregiving role enabling them to take time to use formal health and social services that were focused on health promotion. For example, one caregiver said:

Oh yeah, we had a very supportive system with our closer friends they were calling all week, wanted to make sure how the progress was, and you know if there’s anything they could do for us and that type of thing. So yeah, that was good. (SCG 17)

It was not easy to discern why some caregivers were well-supported by their social networks while others were less supported. But those caregivers who had strong, close networks prior to the stroke appeared to fare better than those who were more insular or constrained by their life situation. Another caregiver said, “the social support from the community as a whole, was outstanding.” (SCG 07) Providers also alluded to the willingness and availability of family members to provide respite as a valuable part of supporting the caregiver. This respite gave the caregiver an opportunity for social engagement outside of their caregiving role. For example, one provider said, “where I see things more successfully done is, is there family around that can pitch in and give the caregiver some help and sometimes just respite a few days away is important.” (HP 14).

## Discussion

The aim of the current study was to explore the factors that influence stroke caregivers’ use of formal health and social services. To our knowledge, this is the first Canadian study to explore caregiver service access and use beyond the immediate post-acute stroke time, six months to five years post-stroke. Furthermore, the study findings provide a long-term perspective (mean 30 months, *SD* = 14.0 months, after stroke) on service use and the stroke recovery trajectory that is underrepresented in the stroke literature to date [61]. The research also included the perspectives of multiple health providers which further substantiated the study findings and fostered greater understanding of service provision to stroke caregivers.

The current study contributes important new findings to our understanding of the impact that financial, health and social factors have on caregivers’ ability to make use of supportive services. The study findings also extend the literature through the use of a conceptual framework [45] that was used to develop and guide this qualitative study and to situate the study findings. The aforementioned results from the current study help explain the needs-services gap of the RC – MMC framework indicating how various factors beyond service provision impact service use by stroke caregivers.

An important finding of the current study was that finances and availability of transportation influenced stroke caregivers’ access to and use of services. While other studies conducted in the USA also determined that finances are a limiting factor to access and use of services [27], this study highlights the importance of this factor even within a publicly funded health system such as in Canada. In the current study, loss of employment affected caregivers’ household income in two ways: first, when caregivers themselves had to reduce or quit their work to fulfill their caregiver role, and second, when the stroke survivor was the primary income earner and could no longer work. Prior studies lend support to the finding that decrease or loss of employment is a factor that can exacerbate the burden experienced by caregivers [15, 27, 62], but these studies did not discuss the connection between employment and ability to access or use services. Although the financial costs associated with caregiving are well documented [63–65], the current study enhances understanding of how limited finances can negatively impact the ability of caregivers to access and use services. Furthermore, our study also determined that caregivers experienced challenges in finding information about financial subsidies, such as disability support, day programs or equipment subsidies which negatively impacted their use of services. These challenges included: needing access to computers, not knowing whom to approach for assistance, and simply not knowing that programs existed or were intended to support them, findings that are corroborated by prior studies [33, 66, 67]. A systematic literature review of challenges, satisfactions and coping strategies of stroke caregivers also mentioned finances as a common challenge, but did not make the explicit connection to reduced access to and use of services [67].

Caregivers and health providers alike spoke to the challenges caregivers faced in managing their own health while engaging in a prolonged caregiving role. Integral to their health management was the ability to access services such as family physicians, counsellors, and health promotion programs. Unfortunately the burden of caregiving frequently left caregivers little time and energy for self-care and combined with a lack of support for the stroke survivor, often meant that their health was neglected, as also supported by the literature [68]. The findings of this study highlight that the availability of respite and concurrent programs that would enable stroke survivors and caregivers to simultaneously engage in health supporting programs could make critical contributions to caregiver health.

A key study finding was that caregivers struggled to trust the health providers who were providing care to the stroke survivor in their absence. Some of this lack of trust stemmed from limited continuity in service provision regarding homecare. This lack of trust meant that caregivers spent more time explaining the needs of the stroke survivor to the homecare worker rather than benefiting from the respite. In addition, caregivers also identified service unreliability as a source of stress for them. If they were unsure of who would be coming to care for the stroke survivor, then they were less likely to leave the house. Caregivers also discussed their fear and anxiety related to leaving the stroke survivor alone after their stroke. Their fears stemmed from concern that the stroke survivor might experience another stroke in their absence or behave in a manner that would threaten their health or safety. This finding is consistent with the literature, where caregivers report their fears associated with the stroke survivor experiencing another stroke [33, 69, 70]. The findings of the current study add to the literature by highlighting how lack of trust in health providers and in the stabilized health of the stroke survivor hindered caregivers from using services for their own health.

The study findings call attention to an overall lack of information and availability of services to support caregivers in community settings. Challenges varied from issues accessing services such as disability or other financial supports to simply not knowing what existed to support them, as corroborated by the literature [71]. Addressing these challenges may require the development of roles such as stroke navigators, which currently mainly exist within acute care settings. Additional areas for growth and improvement include the need for development and improvement of mHealth tools that can support stroke caregivers [72]. Digital applications such as smartphone apps, email and internet-based tools have the potential to facilitate service delivery to caregivers and stroke survivors. However, more research is needed to better understand feasibility and acceptability of these tools across a range of socioeconomic, cultural and geographic parameters [73, 74].Inherent in such tools is also the recognition that caregivers must have access to technology as well as support and training to optimize use of such services. Past research suggests that caregivers have diverse abilities and preferences for supportive telehealth technologies, emphasizing the need for a variety of options to support and foster service use by this population [75]. Further, SARS-CoV-2 (COVID-19) has both highlighted the requirement of digital applications to support caregivers and stroke survivors while also providing the needed catalyst to facilitate their uptake [76].

Importantly, the current study also demonstrates the influence of caregivers’ social networks on their use of services. While it is well documented in the literature that caregivers can experience a change in their social networks because of assuming a caregiving role [18, 20, 29, 35], there is limited literature on the consequences of diminished social networks on caregivers’ service use. Some caregivers in the current study described being abandoned by their social networks such that caregivers were subsequently forced to access respite services to manage basic household tasks, complete groceries and banking or attend their own health appointments. The literature describes the loss of stroke caregivers’ social networks related to their caregiving responsibilities [22, 29]. However, this is the first Canadian study to describe how caregivers diminished social networks can lead to increased use of formal services.

### Implications

Our study findings have several implications for Canadian healthcare policy and programs that may be relevant world-wide. Many community-based services that would benefit caregivers are not covered financially and must be paid out-of-pocket. The occurrence of a stroke can add to the financial constraints experienced by stroke survivors and their caregivers, making the use of community-based services unaffordable for some. These effects can be especially strong for older stroke survivors and their caregivers living on a fixed post-retirement income. Going forwards there is a need for readily available and accessible health and social services to support caregivers in the community setting [17, 23, 77]. Improving access to services for these survivors and caregivers would require increasing access to financial aid, possibly through enhanced subsidies or income tax reductions, or by increasing the list of insured community-based services. Changes to healthcare policy to recognize stroke caregivers as an integral part of the circle of care for stroke survivors could also ensure service accessibility for caregivers.

At a healthcare program level, there is increasing recognition of the importance of family-centered care [78] as well as the need to include ongoing assessment and service provision to stroke caregivers [79–81]. Community-based nurses and a variety of allied health professionals (e.g. speech language pathologists, recreation therapists, social workers, occupational therapy, and physiotherapy) have a strong role to play in addressing these healthcare program aspects by supporting, educating and facilitating care and communication among caregivers and stroke survivors [30].

### Strengths and Limitations

A strength of the current study was our rigorous analytic approach, including using multiple data sources such as interviews and ecomaps and using several strategies to ensure the rigor of this qualitative study. Another strength of this study was the inclusion of a broad sample of caregivers and health providers to inform our understanding of the factors influencing their access to and use of formal health and social services within the Canadian context.

One of the study limitations was the use of single interviews that often were conducted a considerable time after the stroke caregiver started using services, thereby necessitating caregivers’ recall over potentially long periods of time. This may have resulted in participants placing more emphasis on recent experiences or potentially erroneously recalling service use experiences. However some study participants were newer (< 18 months post-stroke, *n* = 6) caregivers and therefore less likely to experience recall bias. A longitudinal study design may be better placed to facilitate experiential recall of access and service use over time in stroke caregivers. The study sample included mainly spousal caregivers with a nominal representation of parent stroke caregivers and no adult child caregivers. Therefore, the study findings cannot inform service access and use experiences of adult child caregivers. Every effort should be made to include these important segments of the stroke caregiving population in future research samples. Recruitment strategies such as use of social media or digital media may have facilitated access to these participants. Further, cultural and financial diversity within study participants was limited thereby negatively impacting the study’s ability to increase understanding about cultural or financial impacts on caregivers’ access and use of services. This could potentially be addressed in the future by using stratified sampling techniques based on cultural group, or socioeconomic and geographic boundaries. 

## Conclusion

Providing care to a stroke survivor over prolonged periods of time can negatively impact caregivers’ health and well-being. While some caregivers make use of services intended to support them in their caregiving role; there has been a gap in understanding why some caregivers do not access and use supportive services. Findings from the current study suggest that caregivers face multiple socioeconomic limitations impacting their ability to use services use. Moreover, complex care relationships between the caregiver and the stroke survivor and caregiver and health providers can further constrain caregivers’ access and use of services.

Next steps to help optimize caregivers’ access and use of formal health and social services includes improved inclusion of caregivers at all stages of the stroke caregiving trajectory, particularly beyond the post-acute care period. Increased caregiver engagement with the health system will help foster trust between caregivers and health providers, allow for early identification of caregiver health issues and provide greater opportunity to facilitate supportive service use by caregivers. Health providers are ideally positioned to support these caregivers but often have limited opportunity to engage with caregivers in community settings. A greater onus must be placed on the health system to regularly assess caregivers’ need for services and link them to these services. Increased funding for subsidies and community programs is required to provide support to caregivers and stroke survivors.

**Table 1:** Characteristics of stroke caregivers (*n* = 22)

Variable		*n* (%)
Age (years)^a^		
40 – 49	1 (4.5)	
50 – 59	5 (22.7)	
60 – 69	10 (45.5)	
70 – 79	4 (18.2)	
80 – 89	2 (9.1)	
Sex		
Female	14 (63.6)	
Male	8 (36.4)	
Geographic Location		
Urban	19 (86.4)	
Rural	3 (13.6)	
Household Income ($ CAN)		
≤ 19,999	2 (9.1)	
20,000 – 39,999	6 (27.3)	
40,000 – 59,999	3 (13.6)	
≥ 60,000	8 (36.3)	
Prefer Not to Answer	3 (13.6)	
Employed		
Yes	8 (36.4)	
No	14 (63.6)	
Relationship to Stroke Survivor		
Spouse	19 (86.4)	
Parent	3 (13.6)	
Receiving Formal In-home Services		
Yes	10 (45.5)	
No	12 (54.5)	
Type of Service		
Homecare support^b^	8 (36.4)	
Other	2 (9.1)	

*Notes.* ^a^mean age = 62.9 years, *SD* = 9.7, ^b^via Local Health Integration Networks

**Table 2:** Charecteristics of Stroke Caregivers

Variable	*n* (%)
Duration of Caregiving (months)^a^	
0 – 12	2 (9.1)
13 – 24	8 (36.4)
25 – 36	2 (9.1)
≥ 37	10 (45.5)
Number of MCC (number)^b^	
0 – 1	6 (27.3)
2 – 4	12 (54.5)
5 – 7	3 (13.6)
8 – 10	1 (4.5)

*Notes*. *n* = 22, MCC = multiple chronic conditions, ^a^ $\overline{x }$ = 30.0, *SD* = 14.0, ^b^ $\overline{x }$ = 3.1, *SD* = 2.1

**Table 3:** Characteristics of Stroke Survivors (*n* = 21)

Variable	*n* (%)
Age (years)^a^	
30 – 39	1 (4.7)
40 – 49	1 (4.7)
50 – 59	6 (28.6)
60 – 69	8 (38.1)
70 – 79	4 (19.0)
80 – 89	1 (4.7)
Sex	
Female	8 (38.1)
Male	13 (61.9)
Number of MCC (number)^b^	
0 – 1	1 (4.7)
2 – 4	11 (52.4)
5 – 7	8 (38.1)
8 – 10	1 (4.7)

**Table 4:** Characteristics of Health Providers (*n* = 18)

Variable	*n* (%)
Type of Health Provider	
Registered Nurse or Clinical Nurse Specialist	5 (27.8)
Physician (General or Specialist)	3 (16.7)
Occupational Therapist	1 (5.6)
Speech Language Pathologist (SLP)	2 (11.1)
Social Worker	3 (16.7)
Therapeutic Recreationist	1 (5.6)
Kinesiologist	1 (5.6)
Personal Support Worker (PSW)	2 (11.1)
Sex	
Female	14 (77.8)
Male	4 (22.2)
Age (years)	
≤ 30	3 (16.7)
31 – 40	7 (38.8)
41 – 50	1 (5.6)
51 – 60	5 (27.8)
≥ 61	2 (11.1)
Years Employed in Field (years)	
≤ 3	3 (16.7)
4 – 10	4 (22.2)
11 – 15	1 (5.6)
16 – 20	3 (16.7)
≥ 21	7 (38.8)
Level of Education	
Diploma or Certificate	5 (27.8)
Undergraduate Degree	3 (16.7)
Graduate Degree	10 (55.6)
Years in Current Position (years)	
≤ 3	9 (50.0)
4 – 10	5 (27.8)
≥ 21	4 (22.2)

**Table 5:** Main Themes and Sub-Themes

Main Theme with Exemplary Quote	Corresponding Sub-theme
Finances and Transportation “*You have to pay; well, we can’t afford $50 or $75 twice a week*”	Paying out-of-pocket for services Reduction in income and employment Navigating financial assistance systems Access to subsidized transportation
Challenged to take care of my own health “*How can I find the time?*”	Limited time and coping ability Few services available Limited system wide attention on importance of caregiver health
Trust “*He’s breathing, but is that good enough?*”	Difficulty trusting that I can leave my family member alone Struggling to trust providers caring for my family member
Limited information and lack of suitable services “*There’s a year-long wait list*”	Insufficient information on services Challenges with service availability
Social support networks “*They deserted us; they just don’t come around*”	Social networks were avoidant Social networks rallied

## Supplementary Information

**Additional file 1.**

**Additional file 2. **

**Additional file 3.**

## Data Availability

The datasets used and/or analysed during the current study are available from the corresponding author on reasonable request.
