# Supplementary material for: Factors impacting the access and use of formal health and social services by caregivers of stroke survivors: an interpretive description study
Source: BMC Health Serv Res. 2022 Apr 1;22:433. doi: 10.1186/s12913-022-07804-x (PMC8975449; doi:10.1186/s12913-022-07804-x)
Supplement: Supplementary file 1 — Additional file 1. [file 12913_2022_7804_MOESM1_ESM.docx]

Appendix A

Questions to guide in-depth interviews with stroke caregivers

***Overarching Questions***

- Can you tell me about your life as a caregiver?
- Can you tell me about a time when you felt supported in your role as a caregiver?
- Can you tell me about some of the challenges you have faced as a caregiver?
- Can you tell what has been helpful to you in managing these challenges?
- Can you tell me how being a caregiver has affected your life?
- Can you tell me what an average day looks like for you in your role as a caregiver?
- Can you tell me how being a caregiver of an adult with stroke has affected your health?
- Can you tell me how you would like to be supported in managing your own health and in your caregiving role?
- Can you tell me how your experience as a caregiver has changed over time?
- What is it like managing the complexity of MCC (in adult with stroke) as a caregiver?

***Caregiving Role***

- Can you tell me what qualities you bring to your caregiving? Prompt – Would you say you have specific skills or abilities?
- Can you tell me about what supports you provide?
- What are qualities that you bring to caregiving?
- How has caregiving changed you? What skills have you learned?
- What do you look forward to?

***Social Support***

- Can you explain what social supports you have in your life?
- How do these people make a difference in your life?
- Can you tell me about how being a caregiver has affected your social circle?

***Adult with Stroke***

- Can you explain how your relationship with your (stroke survivor /husband/wife/mother/father/other) has been affected by becoming a caregiver?

***Health System/Community Resources***

- Can you tell me about your experience in accessing and using services?
- Can you tell me about a time when you needed/wanted/used a service, either to support you in managing your own health or in your caregiving role?
- Have you accessed formal services to support you in your caregiving role?
- What makes it easy to use services?
- What makes it hard to use services?
- What recommendations do you have regarding services?
- What happened? How did you know about it? Why did you need it? What was going on in your life at the time?
- Can you explain what sort of health or social services you currently receive in your role as a caregiver?
- Can you tell me about the things you consider when choosing to use services?
- Can you summarize what services would help you as a caregiver?

***Need-Services Gap***

- Can you tell me about a time when you tried to access services?
- Can you tell me about what affects your decision to use services?
- Would you be willing to or are you currently paying for privately funded services?
- Are there services that are available to you but you do not use? For what reasons? (e.g. distance, cost of parking, work constraints)

***Health***

- Can you tell me about your health?
- Can you discuss how your health has been affected by your role as a stroke caregiver?
- Are there changes you would like to make to improve or maintain your health?
- Are there geographical constraints on your ability to seek/obtain services or respite?
- Can you tell me how you would feel supported to continue in your caregiving role?
- How would you like to see the health system assist you in your caregiving role?
- What services would you like to be able to use that are not currently available to you?

***Contextual Factors – Economic/Social/Physical***

- Can you tell me about something in your life that has had the most influence on your role as a caregiver?
