# Supplementary material for: Factors impacting the access and use of formal health and social services by caregivers of stroke survivors: an interpretive description study
Source: BMC Health Serv Res. 2022 Apr 1;22:433. doi: 10.1186/s12913-022-07804-x (PMC8975449; doi:10.1186/s12913-022-07804-x)
Supplement: Supplementary file 3 — Additional file 3. [file 12913_2022_7804_MOESM3_ESM.docx]

The data that support the findings of this study are available on request from the corresponding author, [AG]. The data are not publicly available due to their containing information that could compromise the privacy of research participants.
