# Supplementary material for: Factors impacting the access and use of formal health and social services by caregivers of stroke survivors: an interpretive description study
Source: BMC Health Serv Res. 2022 Apr 1;22:433. doi: 10.1186/s12913-022-07804-x (PMC8975449; doi:10.1186/s12913-022-07804-x)
Supplement: Supplementary file 2 — Additional file 2. [file 12913_2022_7804_MOESM2_ESM.pdf]

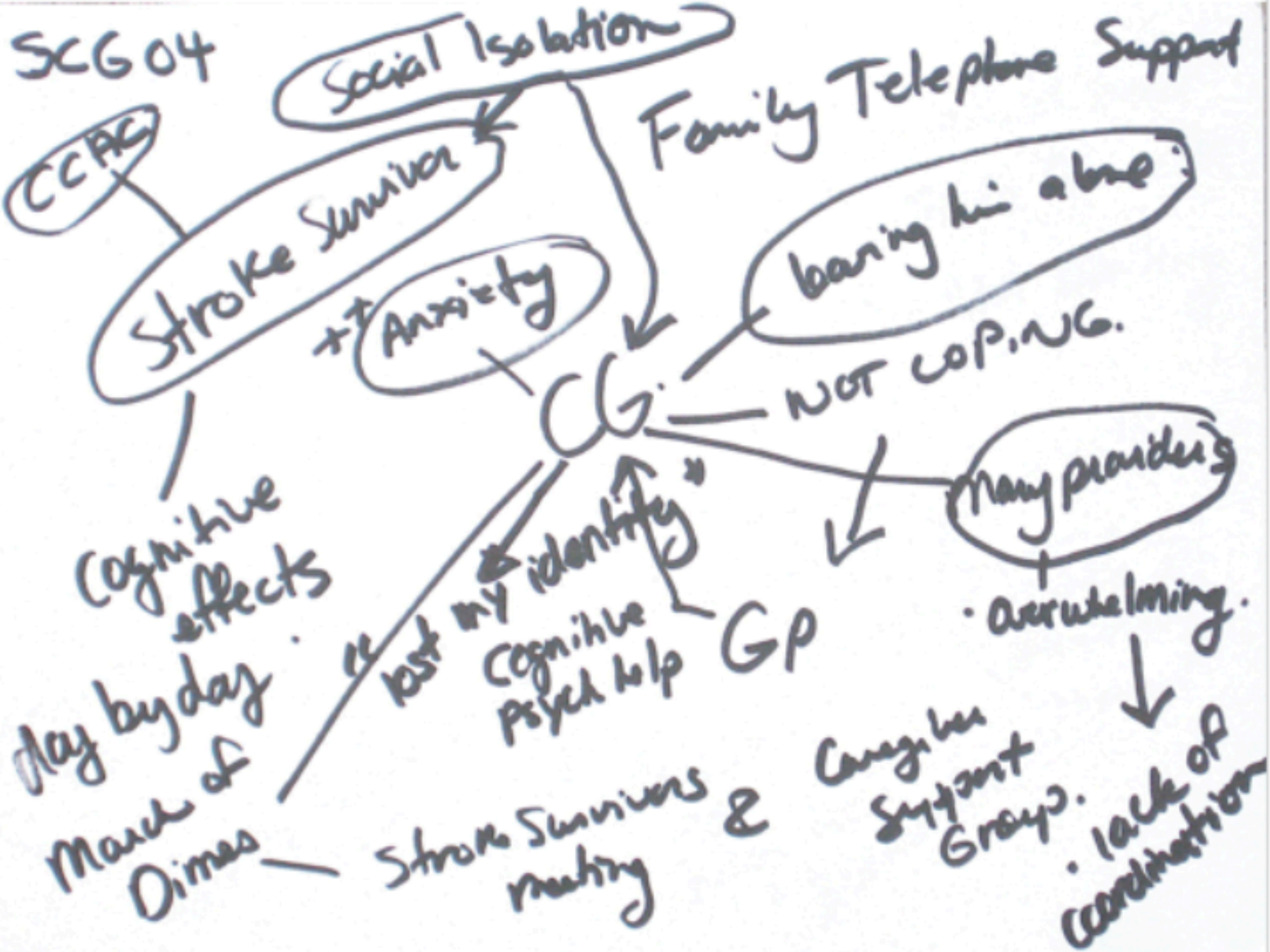
SCG04
Social Isolation
CCAC
Stroke Survivor
Anxiety
Family Telephone Support
leaving him alone
CG
NOT COPING.
many providers
· overwhelming.
↓
· lack of coordination
cognitive effects
day by day.
"lost my identity"
cognitive psych help
GP
March of Dimes
Stroke Survivors meeting
&
Caregiver Support Group.
